# Supplementary material for: Visual barcodes for clonal-multiplexing of live microscopy-based assays
Source: Nat Commun. 2022 May 18;13:2725. doi: 10.1038/s41467-022-30008-0 (PMC9117331; doi:10.1038/s41467-022-30008-0)
Supplement: Supplementary file 1 — Supplementary Information [file 41467_2022_30008_MOESM1_ESM.pdf]

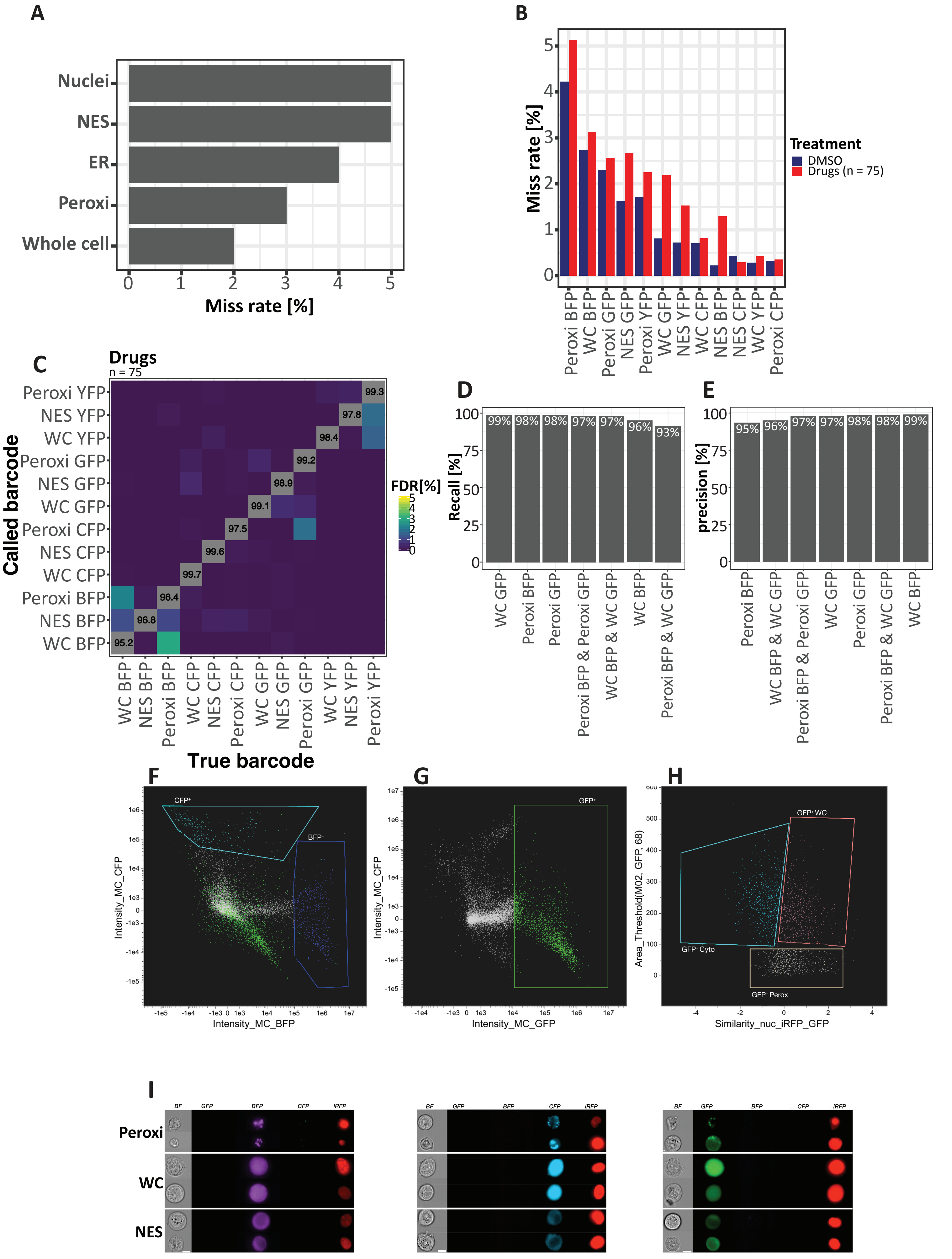

A
Nuclei
NES
ER
Peroxi
Whole cell
Miss rate [%]
B
Miss rate [%]
Treatment
DMSO
Drugs (n = 75)
Peroxi BFP
WC BFP
Peroxi GFP
NES GFP
Peroxi YFP
WC GFP
NES YFP
WC CFP
NES BFP
NES CFP
WC YFP
Peroxi CFP
C
Drugs
n = 75
Called barcode
True barcode
FDR[%]
Peroxi YFP
NES YFP
WC YFP
Peroxi GFP
NES GFP
WC GFP
Peroxi CFP
NES CFP
WC CFP
Peroxi BFP
NES BFP
WC BFP
99.3
97.8
98.4
99.2
98.9
99.1
97.5
99.6
99.7
96.4
96.8
95.2
D
Recall [%]
99%
98%
98%
97%
97%
96%
93%
WC GFP
Peroxi BFP
Peroxi GFP
Peroxi BFP & Peroxi GFP
WC BFP & WC GFP
WC BFP
Peroxi BFP & WC GFP
E
precision [%]
95%
96%
97%
97%
98%
98%
99%
Peroxi BFP
WC BFP & WC GFP
Peroxi BFP & Peroxi GFP
WC GFP
Peroxi GFP
Peroxi BFP & WC GFP
WC BFP
F
CFP+
BFP+
Intensity_MC_CFP
Intensity_MC_BFP
G
GFP+
Intensity_MC_CFP
Intensity_MC_GFP
H
GFP+ WC
GFP+ Cyto
GFP+ Perox
Area_Threshold(M02, GFP, 68)
Similarity_nuc_iRFP_GFP
I
BF
GFP
BFP
CFP
iRFP
Peroxi
WC
NES

J

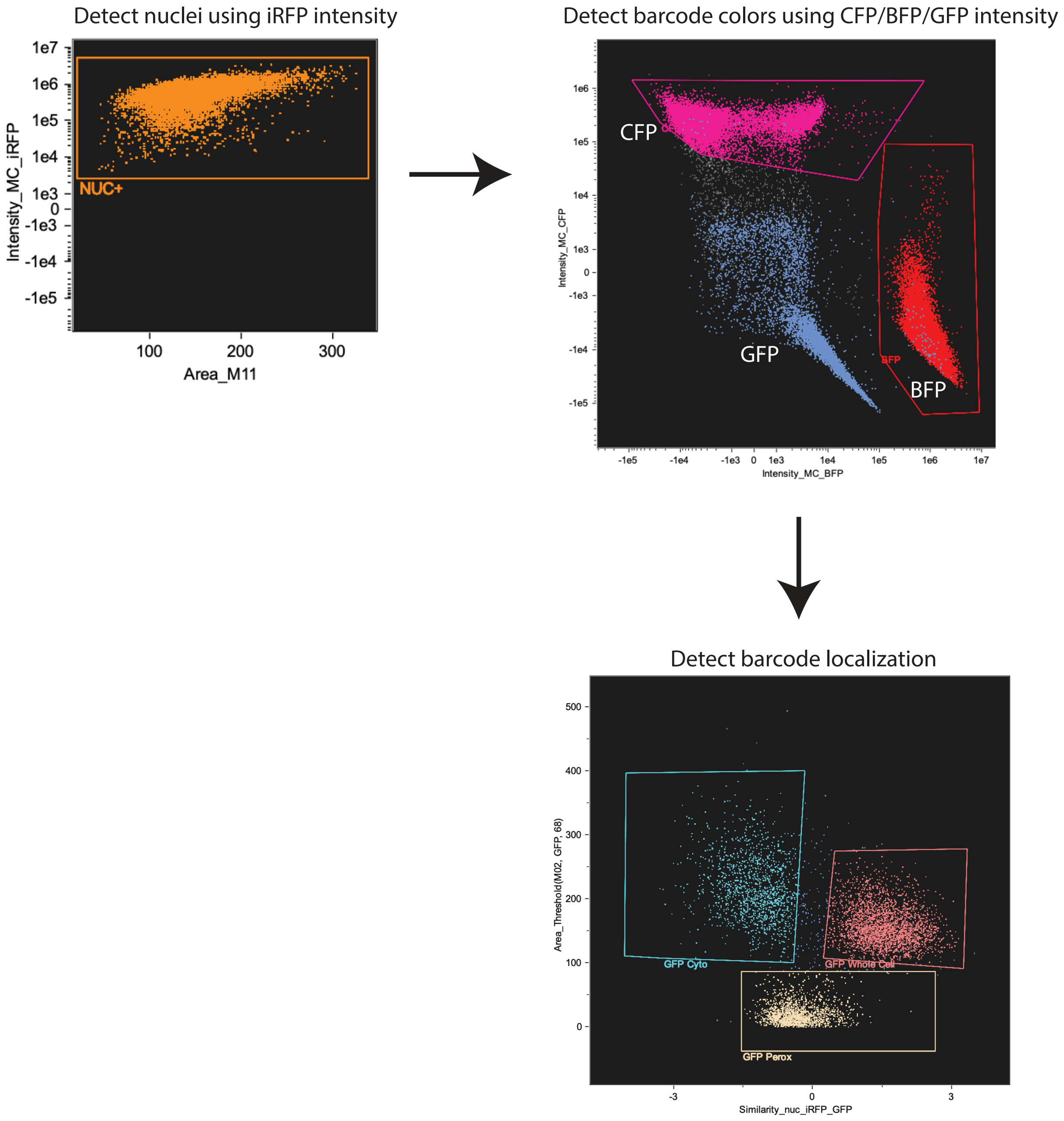
Detect nuclei using iRFP intensity
NUC+
Intensity_MC_iRFP
Area_M11
1e7
1e6
1e5
1e4
1e3
0
-1e3
-1e4
-1e5
100
200
300
Detect barcode colors using CFP/BFP/GFP intensity
CFP
GFP
BFP
Intensity_MC_CFP
Intensity_MC_BFP
Detect barcode localization
GFP Cyto
GFP Whole Cell
GFP Perox
Area_Threshold(M02, GFP, 68)
Similarity_nuc_iRFP_GFP
500
400
300
200
100
0
-3
0
3

Supp Figure 1. Multiplexing cell lines for live imaging applications using visual barcodes. A. Barcode detection miss rate for the five CFP localizations in A375 cells. **B.** Miss rate percentage for all 12 A375 clones with visual barcodes treated with DMSO (Blue, n = 39) or drugs (Red, n = 75). **C.** Average Barcode calling false detection rate for all 12 visual barcoded clones of the A375 cell line treated with a library of 75 drugs. Numbers in the diagonal represent the average precision (%) for each barcode. **D-E.** Bar plots showing recall and precision of barcodes with dual color and localization of the FP. **F-H.** Scatter plots showing the separation of nine A375 clones with visual barcodes by the ImageStream system according to their fluorescent color and localization. The separation by localization is only demonstrated for GFP positive clones (**H**). **I.** Representative images from ImageStream of all nine clones. Two cells from each clone are presented. Scale bar in (**I**) 7µM. **J.** Cells were first gated according to their area (in μm^2) of the iRFP staining. Next, cells were divided to CFP+, BFP+ and GFP+ according to their corresponding intensities. Last, to identify the 3 cell morphologies, two features were calculated for each of the FPs used: Area of the highest intensity pixels using the Threshold mask, and the similarity feature (a measure of the degree to which two images are linearly correlated, calculated as log transformed Pearson's Correlation Coefficient) calculated between each FP and the iRFP signal. Plotting these features on a bi-variate plot gave a clear distinction of the 3 morphologies.

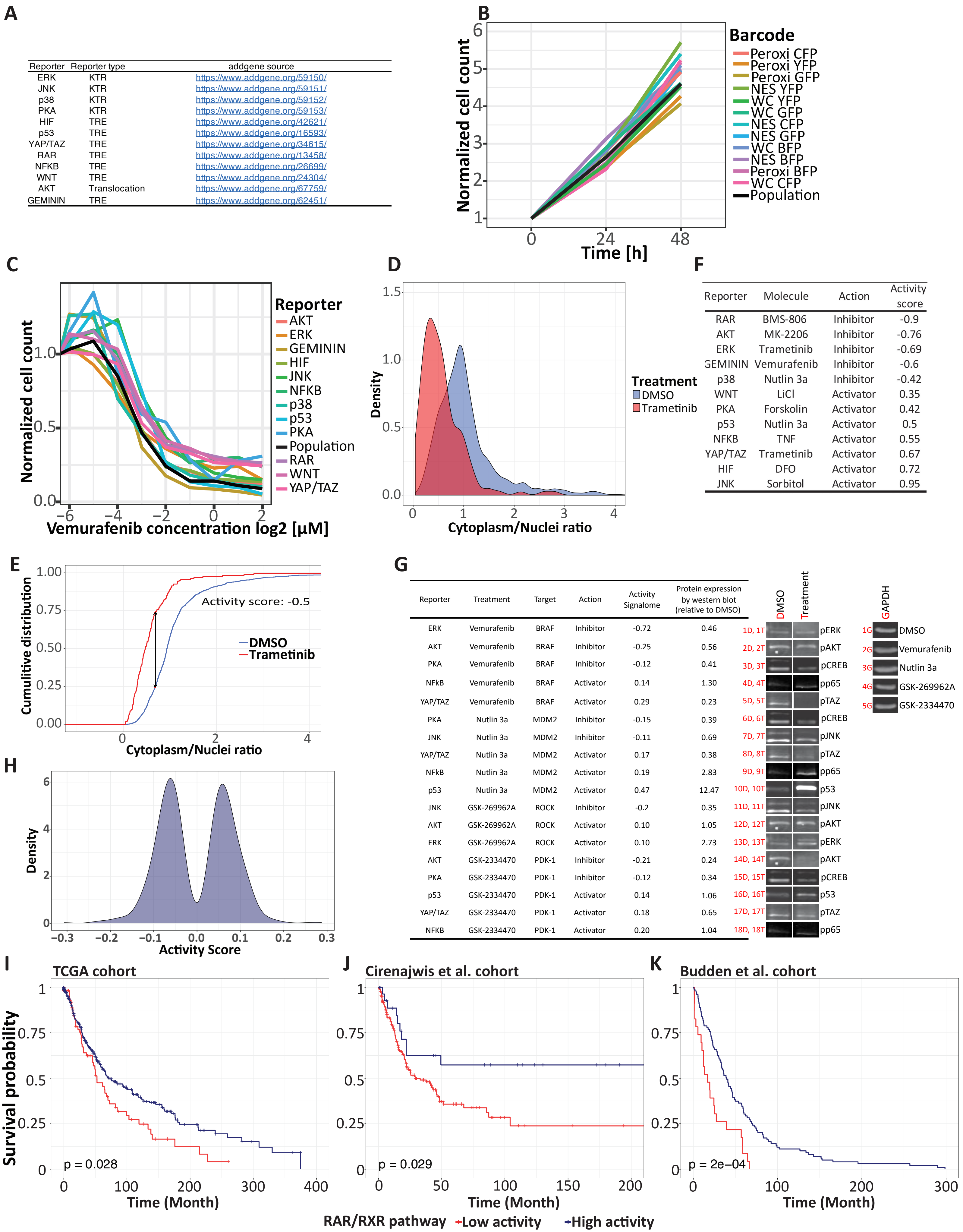

A

Reporter	Reporter type	addgene source
ERK	KTR	https://www.addgene.org/59150/
JNK	KTR	https://www.addgene.org/59151/
p38	KTR	https://www.addgene.org/59152/
PKA	KTR	https://www.addgene.org/59153/
HIF	TRE	https://www.addgene.org/42621/
p53	TRE	https://www.addgene.org/16593/
YAP/TAZ	TRE	https://www.addgene.org/34615/
RAR	TRE	https://www.addgene.org/13458/
NFKB	TRE	https://www.addgene.org/26699/
WNT	TRE	https://www.addgene.org/24304/
AKT	Translocation	https://www.addgene.org/67759/
GEMININ	TRE	https://www.addgene.org/62451/

F

Reporter	Molecule	Action	Activity score
RAR	BMS-806	Inhibitor	-0.9
AKT	MK-2206	Inhibitor	-0.76
ERK	Trametinib	Inhibitor	-0.69
GEMININ	Vemurafenib	Inhibitor	-0.6
p38	Nutlin 3a	Inhibitor	-0.42
WNT	LiCl	Activator	0.35
PKA	Forskolin	Activator	0.42
p53	Nutlin 3a	Activator	0.5
NFKB	TNF	Activator	0.55
YAP/TAZ	Trametinib	Activator	0.67
HIF	DFO	Activator	0.72
JNK	Sorbitol	Activator	0.95

G

Reporter	Treatment	Target	Action	Activity Signalome	Protein expression by western blot (relative to DMSO)
ERK	Vemurafenib	BRAF	Inhibitor	-0.72	0.46
AKT	Vemurafenib	BRAF	Inhibitor	-0.25	0.56
PKA	Vemurafenib	BRAF	Inhibitor	-0.12	0.41
NFkB	Vemurafenib	BRAF	Activator	0.14	1.30
YAP/TAZ	Vemurafenib	BRAF	Activator	0.29	0.23
PKA	Nutlin 3a	MDM2	Inhibitor	-0.15	0.39
JNK	Nutlin 3a	MDM2	Inhibitor	-0.11	0.69
YAP/TAZ	Nutlin 3a	MDM2	Activator	0.17	0.38
NFkB	Nutlin 3a	MDM2	Activator	0.19	2.83
p53	Nutlin 3a	MDM2	Activator	0.47	12.47
JNK	GSK-269962A	ROCK	Inhibitor	-0.2	0.35
AKT	GSK-269962A	ROCK	Activator	0.10	1.05
ERK	GSK-269962A	ROCK	Activator	0.10	2.73
AKT	GSK-2334470	PDK-1	Inhibitor	-0.21	0.24
PKA	GSK-2334470	PDK-1	Inhibitor	-0.12	0.34
p53	GSK-2334470	PDK-1	Activator	0.14	1.06
YAP/TAZ	GSK-2334470	PDK-1	Activator	0.18	0.65
NFKB	GSK-2334470	PDK-1	Activator	0.20	1.04

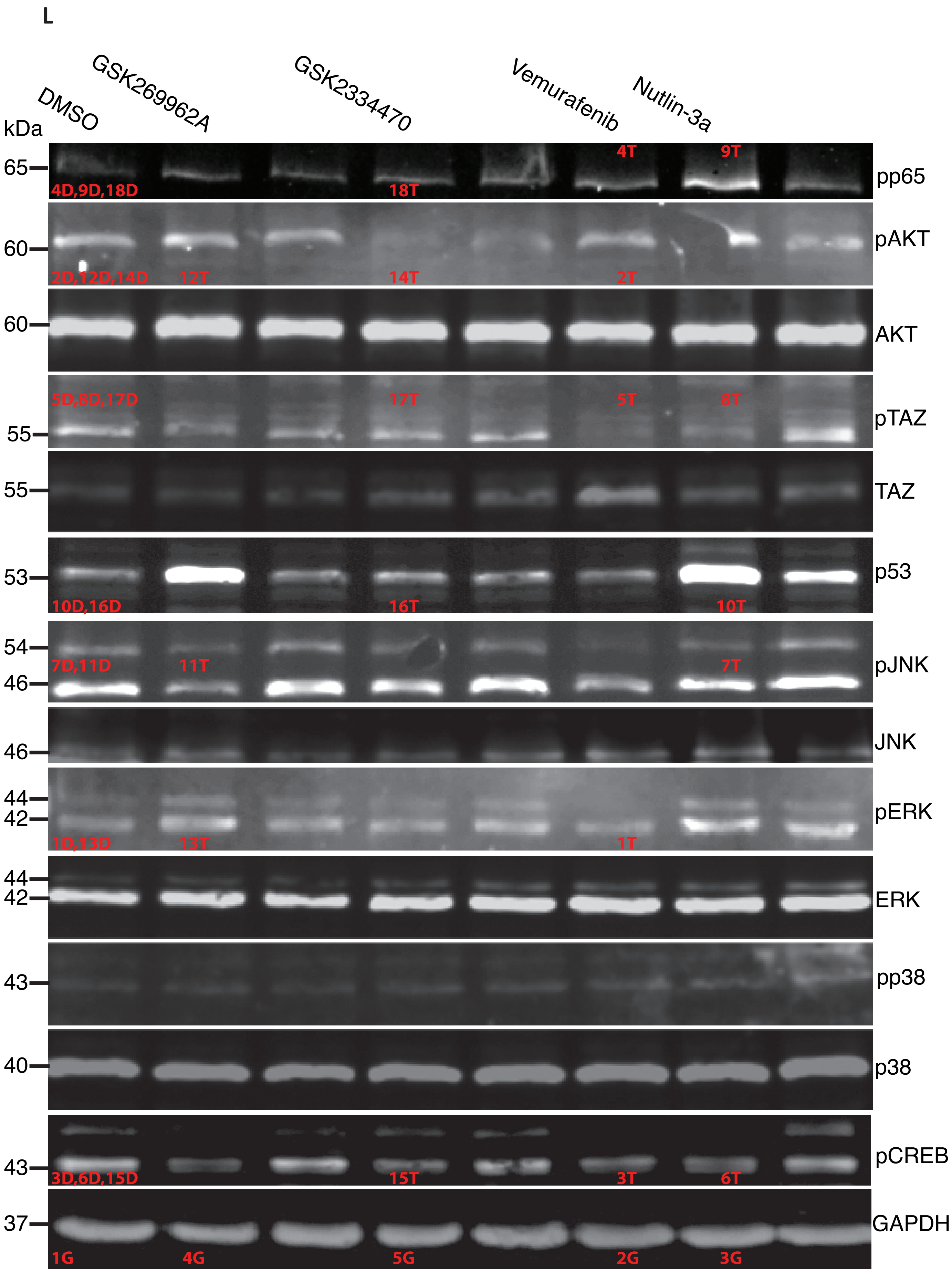
L
DMSO
GSK269962A
GSK2334470
Vemurafenib
Nutlin-3a
kDa
65
60
60
55
55
53
54
46
46
44
42
44
42
43
40
43
37
pp65
pAKT
AKT
pTAZ
TAZ
p53
pJNK
JNK
pERK
ERK
pp38
p38
pCREB
GAPDH
4T
9T
4D,9D,18D
18T
2D,12D,14D
12T
14T
2T
5D,8D,17D
17T
5T
8T
10D,16D
16T
10T
7D,11D
11T
7T
1D,13D
13T
1T
3D,6D,15D
15T
3T
6T
1G
4G
5G
2G
3G

Supp Figure 2. Generating the 'Signalome' reporter cell lines. A. Reporter type and addgene source for each of the 12 reporters used for the Signalome cell lines. **B.** Growth rate of all 12 visual barcode clones and their parental A375 cell line. The parental cell line was labeled with GFP positive lentivirus to facilitate a more accurate cell counting. **C.** Dose response curve of all 12 visual barcoded A375 clones and the parental A375 population (GFP) to vemurafenib. **D.** Density plots of ERK reporter activity depicted by the cytoplasm to nuclei ratio of the ERK translocation reporter after 72h of treatment with DMSO (Blue) or trametinib (Red). **E.** Cumulative distribution of ERK reporter activity of the same cells as in (**D**). KS statistics was used to calculate the activity score. The negative sign of the score reflects the lower mean of the trametinib density plot in (**D**) as compared to the mean of the DMSO plot **F.** Validation of the reporters activity by using known activators/inhibitors of all 12 pathways. **G.** Table and blot showing the validation of the reporter activity as measured using the KS test at a single cell level (each reporter was tested three times with similar results) and the bulk protein expression as measured using western blot. Red annotation represents the location of the band in the original western blot in **Supp Figure 2L.** D-DMSO, T-Treatment, G-GAPDH. **H.** Dstribution of the reporter activity scores in the DMSO control wells (n= 841). Using the control wells density function we chose a strict cutoff of ±0.2 that represents less than 0.5% of false positive results (the 0.5 and 99.5 percentiles are -0.207 and 0.171 respectively). **I-K**. Kaplan-Meier plots showing patient survival stratified by RAR/RXR pathway activity as calculated by PathOlogist, based on RNA-Seq data. TCGA (n = 465 patients), Cirenajwis (n = 210 patients), Budden (n = 122 patients). p-values are the result of log-rang test. **2L.** Western blot validation of reporter activity. The experiment was repeated twice with similar results. See methods for drug concentration and antibodies used.

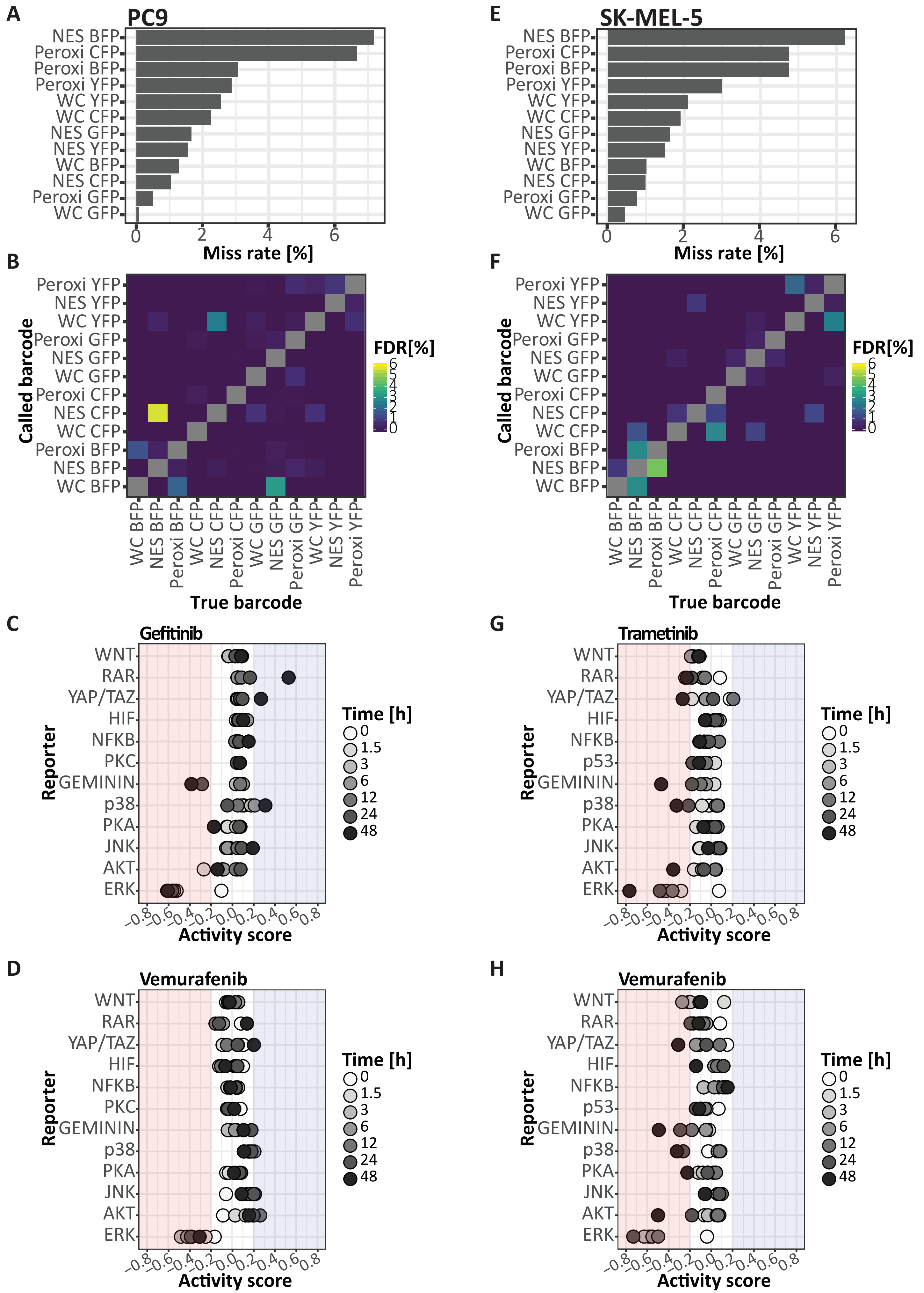

A
PC9
NES BFP
Peroxi CFP
Peroxi BFP
Peroxi YFP
WC YFP
WC CFP
NES GFP
NES YFP
WC BFP
NES CFP
Peroxi GFP
WC GFP
0 2 4 6
Miss rate [%]
B
Called barcode
True barcode
FDR[%]
C
Gefitinib
WNT
RAR
YAP/TAZ
HIF
NFKB
PKC
GEMININ
p38
PKA
JNK
AKT
ERK
Reporter
Activity score
-0.8 -0.6 -0.4 -0.2 0.0 0.2 0.4 0.6 0.8
Time [h]
0
1.5
3
6
12
24
48
D
Vemurafenib
E
SK-MEL-5
F
G
Trametinib
p53
H
Vemurafenib

Supp Figure 3. Creating additional signalome cell lines. A, E. Barcode detection miss rate for PC9 and SK-MEL-5 cell lines respectively. **B, F.** Average barcode false detection rate for all 12 visual clones in both PC9 and SK-MEL-5 cell lines treated with DMSO. **C, D.** PC9 Signalome reporters' activity plot for gefitinib and vemurafenib respectively. **G, H**. SK-MEL-5 Signalome reporters' activity plot for vemurafenib and trametinib respectively.

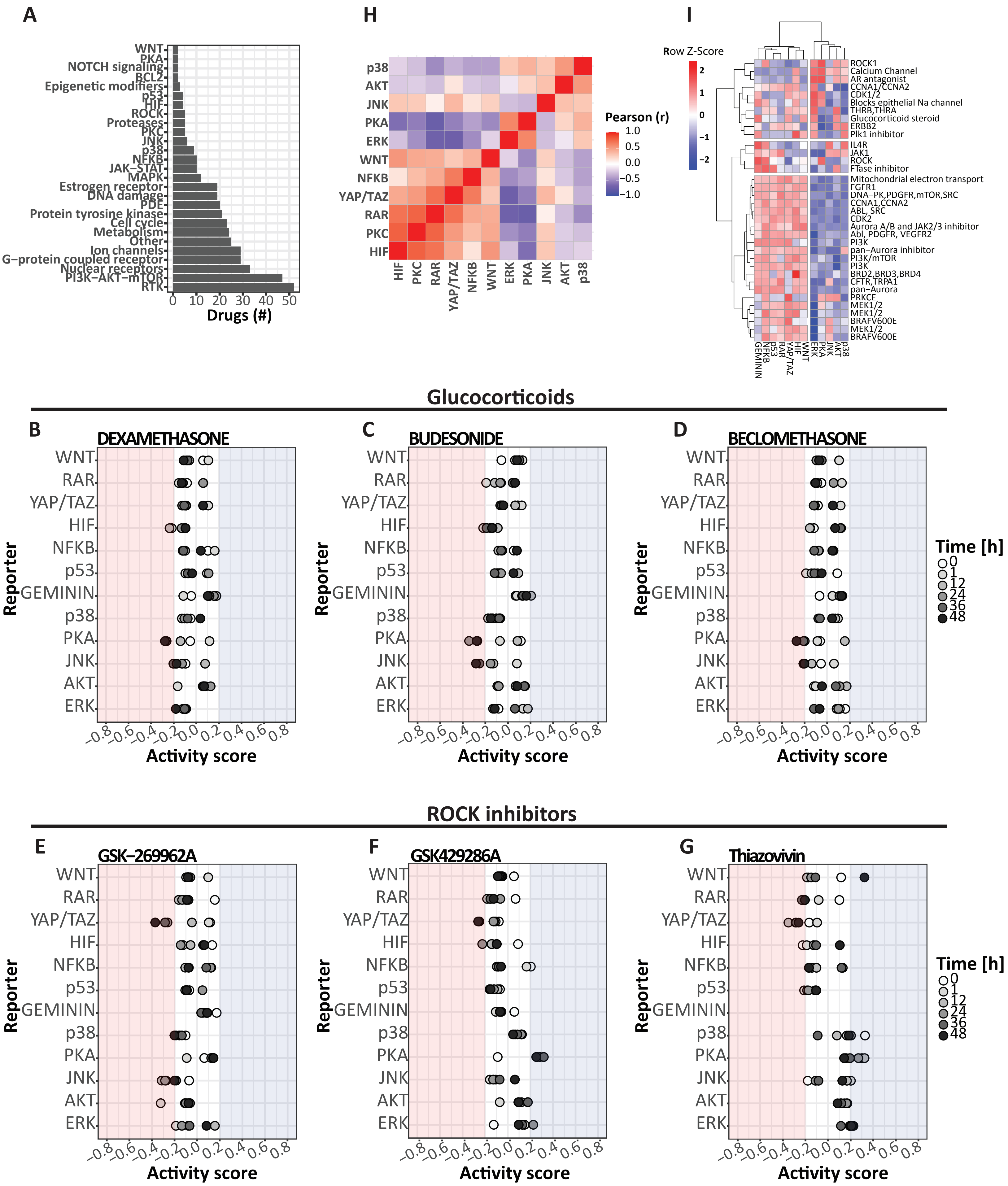
A
WNT
PKA
NOTCH signaling
BCL2
Epigenetic modifiers
p53
HIF
ROCK
Proteases
PKC
JNK
p38
NFKB
JAK-STAT
MAPK
Estrogen receptor
DNA damage
PDE
Protein tyrosine kinase
Cell cycle
Metabolism
Other
Ion channels
G-protein coupled receptor
Nuclear receptors
PI3K-AKT-mTOR
RTK
0 10 20 30 40 50
Drugs (#)
H
p38
AKT
JNK
PKA
ERK
WNT
NFKB
YAP/TAZ
RAR
PKC
HIF
HIF PKC RAR YAP/TAZ NFKB WNT ERK PKA JNK AKT p38
Pearson (r)
1.0
0.5
0.0
-0.5
-1.0
I
Row Z-Score
2
1
0
-1
-2
ROCK1
Calcium Channel
AR antagonist
CCNA1/CCNA2
CDK1/2
Blocks epithelial Na channel
THRB,THRA
Glucocorticoid steroid
ERBB2
Plk1 inhibitor
IL4R
JAK1
ROCK
FTase inhibitor
Mitochondrial electron transport
FGFR1
DNA-PK,PDGFR,mTOR,SRC
CCNA1,CCNA2
ABL, SRC
CDK2
Aurora A/B and JAK2/3 inhibitor
Abl, PDGFR, VEGFR2
PI3K
pan-Aurora inhibitor
PI3K/mTOR
PI3K
BRD2,BRD3,BRD4
CFTR,TRPA1
pan-Aurora
PRKCE
MEK1/2
MEK1/2
BRAFV600E
MEK1/2
BRAFV600E
GEMININ NFKB p53 RAR YAP/TAZ HIF WNT ERK PKA JNK AKT p38
Glucocorticoids
B
DEXAMETHASONE
C
BUDESONIDE
D
BECLOMETHASONE
WNT
RAR
YAP/TAZ
HIF
NFKB
p53
GEMININ
p38
PKA
JNK
AKT
ERK
Reporter
-0.8 -0.6 -0.4 -0.2 0.0 0.2 0.4 0.6 0.8
Activity score
Time [h]
0
1
12
24
36
48
ROCK inhibitors
E
GSK-269962A
F
GSK429286A
G
Thiazovivin

Supp Figure 4. Drugs with similar mechanisms of action cluster together by their effect of signaling pathways. A. Composition of all 422 drugs used in the screen, based on their mechanism of action or target. **B-D.** Signalome reporter activity plots for the glucocorticoids (GCs) dexamethasone, budesonide and beclomethasone respectively. All GCs showed a similar pattern: inhibition of both JNK and PKA reporters. **E-G.** Signalome reporter activity plots for the ROCK inhibitors: GSK269962A, GSK429286A and thiazovivin respectively. All three drugs have a similar pattern which includes mild activation of ERK and PKA together with significant inhibition of YAP/TAZ. **H.** Unsupervised clustered heatmap showing the pairwise correlations (Pearson) between PC9 reporter clones activity scores under 49 active drugs after 48 hours of treatment. **I.** Unsupervised hierarchical clustering of A375 signalome cells treated by 35 active drugs which included the GEMININ reporter. Clustring was according to the reproter activity score.

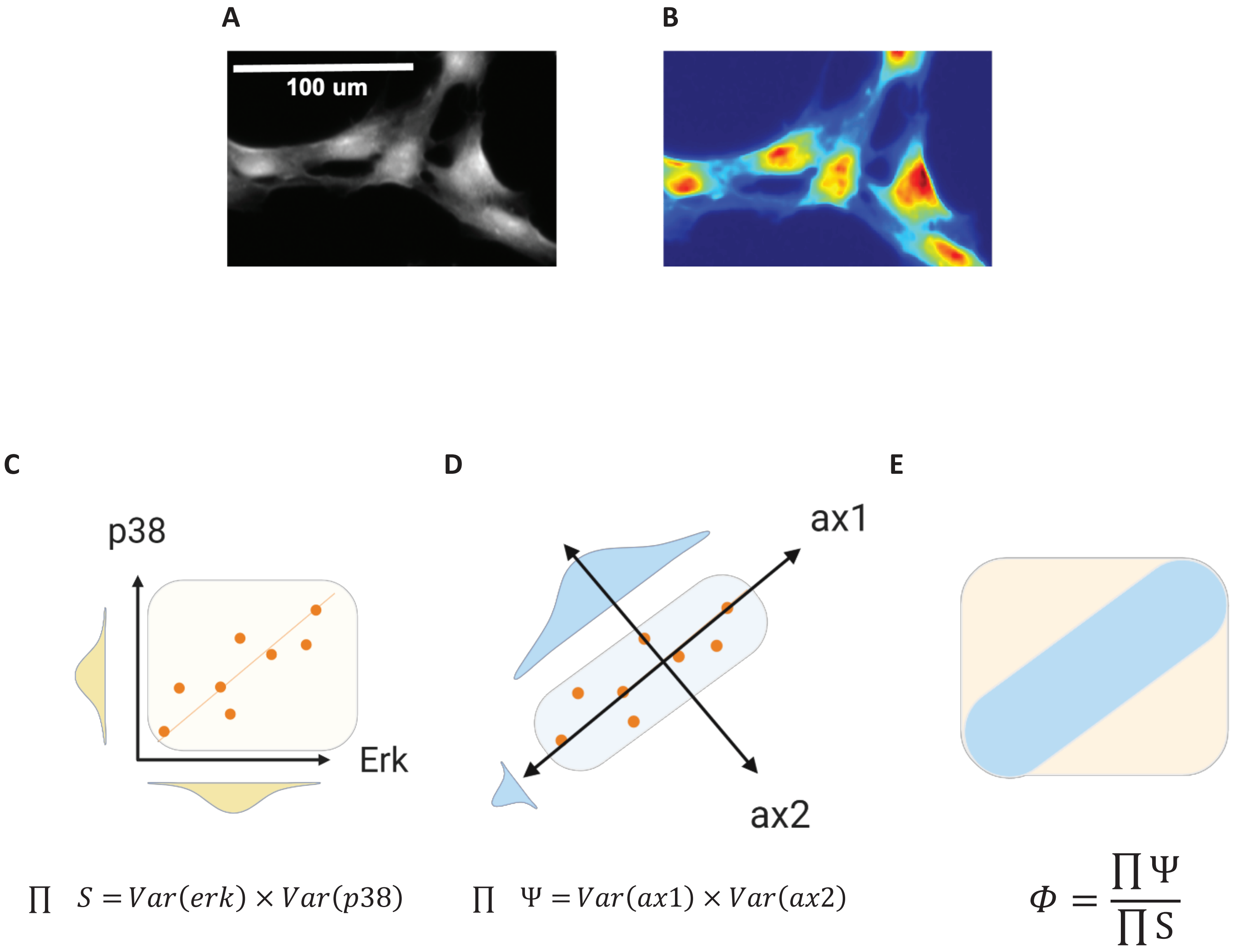
A
100 um
B
C
p38
Erk
∏ S = Var(erk) × Var(p38)
D
ax1
ax2
∏ Ψ = Var(ax1) × Var(ax2)
E
Φ = ∏Ψ / ∏S

Supp Figure 5. Calculating the extent to which principle components of cells that were not exposed to drug treatments represent drug-induced correlations. A. Fluorescence microscopy image of Rpe1 cells stained with AlexaFluor-647-succinimidyl ester. **B.** Heatmap showing intensity of fluorescence signal in (**A**), indicating concentration of cellular proteins. **C, D.** An illustration depicting the correlation of p38 and ERK as represented in two different coorvdinate systems. In the first coordinate system **C.**, axes are defined by the measured activities of p38 and ERK. With the second coordinate system **D.**, axes are defined by performing PCA on cells that were not exposed to drug treatment. The yellow and blue shaded regions represent the area (or volume) that encloses the data in the alternate coordinate systems, which is calculated as the product of the variances. As shown in **E.** The volume enclosing the data is smaller when the coordinates are aligned with linear trends with the dataset. To calculate the extent to which the principle components calculated from measurements on cells that were not exposed to drug treatments represent drug-induced correlations, we compare the product of the variances in the alternate coordinate systems.

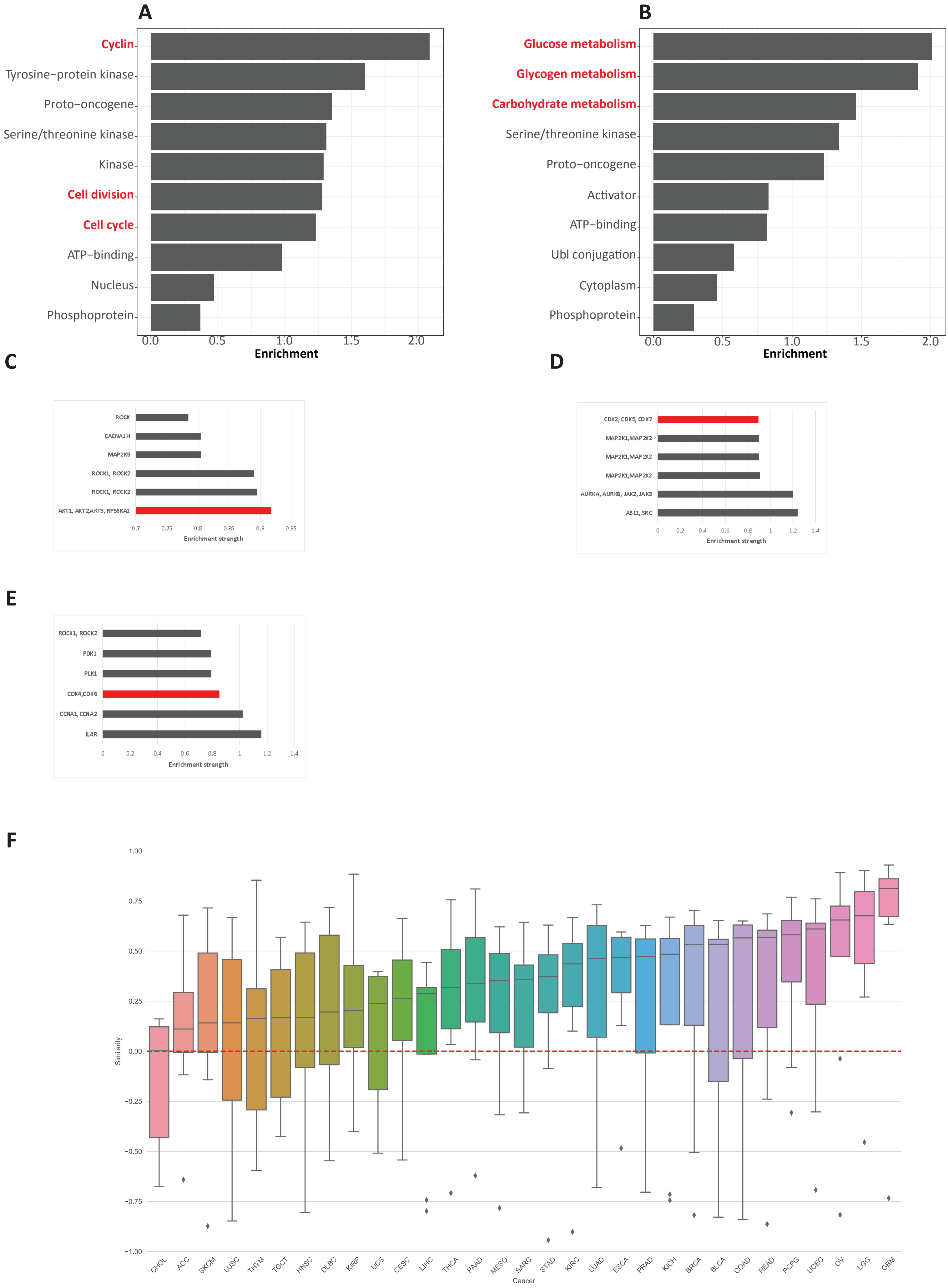

A
Cyclin
Tyrosine−protein kinase
Proto−oncogene
Serine/threonine kinase
Kinase
Cell division
Cell cycle
ATP−binding
Nucleus
Phosphoprotein
0.0 0.5 1.0 1.5 2.0
Enrichment
B
Glucose metabolism
Glycogen metabolism
Carbohydrate metabolism
Serine/threonine kinase
Proto−oncogene
Activator
ATP−binding
Ubl conjugation
Cytoplasm
Phosphoprotein
0.0 0.5 1.0 1.5 2.0
Enrichment
C
ROCK
CACNA1H
MAP2K5
ROCK1, ROCK2
ROCK1, ROCK2
AKT1, AKT2,AKT3, RPS6KA1
0.7 0.75 0.8 0.85 0.9 0.95
Enrichment strength
D
CDK2, CDK5, CDK7
MAP2K1,MAP2K2
MAP2K1,MAP2K2
MAP2K1,MAP2K2
AURKA, AURKB, JAK2, JAK3
ABL1, SRC
0 0.2 0.4 0.6 0.8 1 1.2 1.4
Enrichment strength
E
ROCK1, ROCK2
PDK1
PLK1
CDK4,CDK6
CCNA1, CCNA2
IL4R
0 0.2 0.4 0.6 0.8 1 1.2 1.4
Enrichment strength
F
1.00 0.75 0.50 0.25 0.00 −0.25 −0.50 −0.75 −1.00
Similarity
CHOL ACC SKCM LUSC THYM TGCT HNSC DLBC KIRP UCS CESC LIHC THCA PAAD MESO SARC STAD KIRC LUAD ESCA PRAD KICH BRCA BLCA COAD READ PCPG UCEC OV LGG GBM
Cancer

Supp Figure 6. Characterization of the drug target enriched in each of the clusters. A,B. Drug targets of p38- and p53-signaling state were used as seeds for a functional annotation enrichment analysis. While p53-signaling state (**A**) was found enriched in terms related to cell cycle regulation (cell division), p38-signaling state (**B**) was enriched in terms related to central carbon metabolism and anabolic activity (cell growth). **C-E.** The top drug targets for cluster A (**C**), cluster B (**D**) and cluster C (**E**). **F.** To calculate a similarity score for each TCGA cancer, we calculated the pairwise correlation coefficients (Pearson) between measured activities in the tumor samples and the signalome. The similarity scores were then grouped together in a boxplot to show the distribution of similarities in every pathway for each cancer. The box blots represent the top and bottom quartiles for the distribution with the whiskers showing the extent of the distribution barring outliers (marked by diamonds). A pathway which scores 1 by this measure has the same pattern of correlations and anti-correlations between the given cancer and the signalome. Similarity scores of 1 indicate that all the pathways in the cancer have an identical grouping of correlations and anti-correlations as observed in the signalome. By contrast, scores of zero indicate there is no similarity in the pattern, while negative scores indicate some pathways exhibit inverse patterns in correlations to those observed in the signalome. Median similarity scores indicate the degree to which the pattern of signaling in that cancer was similar to those observed in the signalome. Those above 0 (marked by the red line) indicate that most of the TCGA cancers shared a pattern of correlations with those observed in the signalome. Cholangiocarcinoma (CHOL, n = 30), Adrenocortical carcinoma (ACC, n = 46), Skin Cutaneous Melanoma (SKCM, n = 354), Lung squamous cell carcinoma (LUSC, n = 325), Thymoma (THYM, n = 90), Testicular Germ Cell Tumors (TGCT, n = 118), Head and Neck squamous cell carcinoma (HNSC, n = 346), Lymphoid Neoplasm Diffuse Large B-cell Lymphoma (DLBC, n = 33), Kidney renal papillary cell carcinoma (KIRP, n = 208), Uterine Carcinosarcoma (UCS, n = 48), Cervical squamous cell carcinoma and endocervical adenocarcinoma (CESC, n = 171), Liver hepatocellular carcinoma (LIHC, n = 184), Thyroid carcinoma (THCA, n = 374), Pancreatic adenocarcinoma (PAAD, n = 105), Mesothelioma (MESO, n = 61), Sarcoma (SARC, n = 221), Stomach adenocarcinoma (STAD, n = 392), Kidney renal clear cell carcinoma (KIRC, n = 445), Lung adenocarcinoma (LUAD, n = 362), Esophageal carcinoma (ESCA, n = 126), Prostate adenocarcinoma (PRAD, n = 351), Kidney Chromophobe (KICH, n = 63), Breast invasive carcinoma (BRCA, n = 901), Bladder Urothelial Carcinoma (BLCA, n = 344), Colon adenocarcinoma (COAD, n = 327), Rectum adenocarcinoma (READ, n = 129), Pheochromocytoma and Paraganglioma (PCPG, n = 81), Uterine Corpus Endometrial Carcinoma (UCEC, n = 404), Ovarian serous cystadenocarcinoma (OV, n = 411), Brain Lower Grade Glioma (LGG, n = 427), Glioblastoma multiforme (GBM, n = 205).

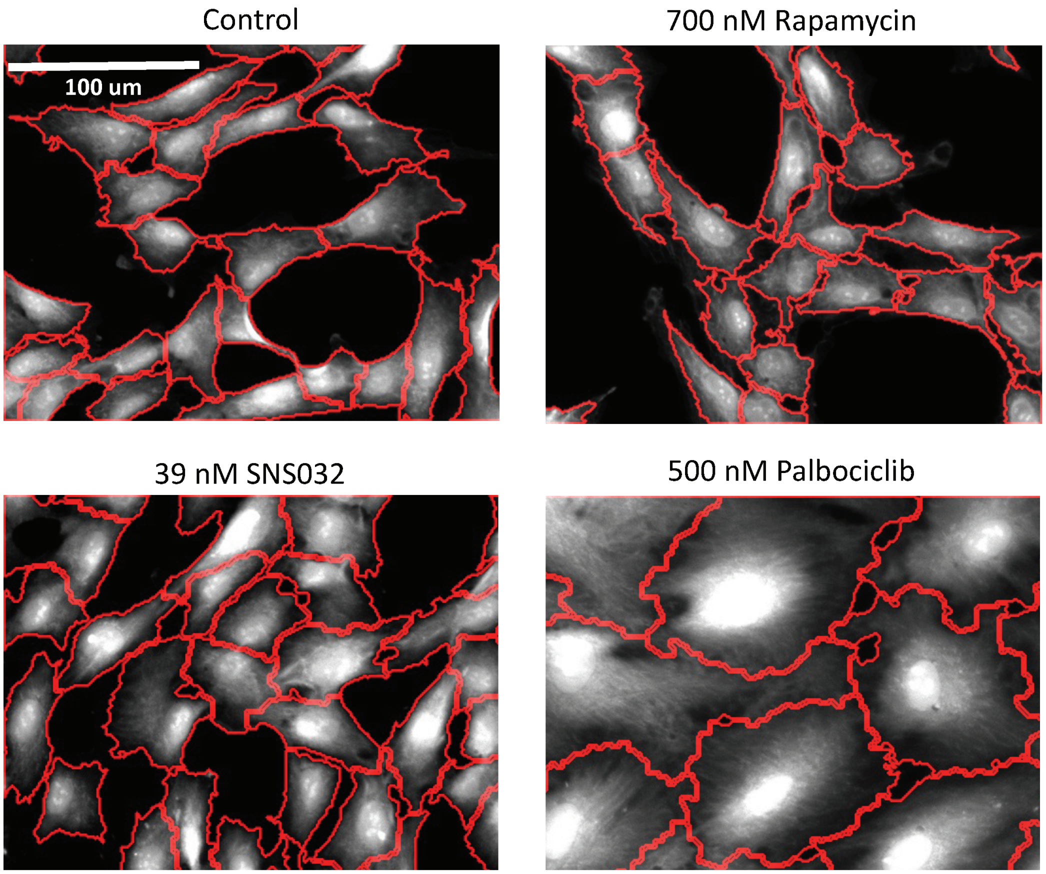

	Fold Change Compared to Control (log_2exp/ctrl)		
**Drug**	**Growth Rate**	**Cell Cycle Length**	**Cell Size**
Control	0	0	0
700 nM Rapamycin	-1.4	0.51	-0.28
39 nM SNS032	-0.12	0.44	0.31
500 nM Palbociclib	0.38	1.6	1.6

Supp figure 7. Incomplete adaptations to selective perturbations of growth or division are dramatically visualized from the constancy of cell size. Rpe1 cells were treated with an mTORC1 inhibitor (rapamycin), cdk1/2 inhibitor (SNS032), or cdk4/6 inhibitor (Palbociclib). Representative widefield fluorescence images (SE-A647 stain) of unperturbed cells and cells after 68 hours of drug treatment are shown. In RPE1 cells, rapamycin treated cells accumulate 60% less mass per unit time. Yet, rather than becoming 60% smaller in size, rapamycin treated cells compensate for the slower rates of growth with longer growth duration (longer cell cycles) resulting in a mere 20% loss of cell size. Similarly, CDK2 inhibited cells (SNS-032) compensate for the 40% longer cell cycles with slower rates of growth resulting in mere 20% increases in cell size. While these results demonstrate the adaptation to perturbations of growth and division with a single pair of inhibitors, a more comprehensive characterization of these was previously reported in Ginzberg et al[21]. The exception to this rule is with CDK4 inhibitors.[21,34] Thus, unlike CDK2 inhibitors, whereby the longer periods of growth are mitigated by slower rates of growth, CDK4 inhibitors promote longer cell cycles and faster rates of growth resulting in significant departure from cell size homeostasis. Intriguingly, in our measurements, CDK4 inhibitors ranked highest in association with cluster C. The table shows $\log_2$ fold-changes are shown relative to the average cell cycle length, growth rate, and cell size of all six control samples (DMSO)
