# Supplementary material for: Visual barcodes for clonal-multiplexing of live microscopy-based assays
Source: Nat Commun. 2022 May 18;13:2725. doi: 10.1038/s41467-022-30008-0 (PMC9117331; doi:10.1038/s41467-022-30008-0)
Supplement: Supplementary file 3 — Description of Additional Supplementary Files [file 41467_2022_30008_MOESM3_ESM.pdf]

Description of Additional Supplementary Files

Supplementary Data 1. Table of drugs and targets included in 75-drug library used to test the robustness of the 12-barcode system under different types of perturbations. After 48 hours of drug treatment, both precision and recall were still very high in almost all drugs, as shown in figure 1F, supplementary figures 1B,C.

Supplementary Data 2. Table of drugs and targets included in 422-drug library used to investigate interdependencies in the cancer signaling, by treating the pooled A375 signalome. Results of this screen are shown in figure 3A, supplementary figures 4I,4B-G.

Supplementary Data 3. Table of full data generated by signalome screen, showing effects of drug library on each measured pathway over the course of the screen. Results are plotted in figure 3A, supplementary figures 4I,4B-G.

Supplementary Data 4. Table of active drugs, targets, and associated clusters identified by signalome screen. Data is plotted in figure 3A-C.

Supplementary Data 5. Table of drugs, targets, and screen results on expanded set of activity reporters including geminin. Data is plotted in supplementary figure 4I.

Supplementary Data 6. Table of active drugs, and screen results obtained using PC9 signalome cell line. Data is plotted in supplementary figure 4H.

Supplementary Data 7. Influence of drug targets on cell size and proliferation rate, as per Liu et al. Data is plotted in figure 6E.
