# Supplementary material for: Visual barcodes for clonal-multiplexing of live microscopy-based assays
Source: Nat Commun. 2022 May 18;13:2725. doi: 10.1038/s41467-022-30008-0 (PMC9117331; doi:10.1038/s41467-022-30008-0)
Supplement: Supplementary file 11 — Reporting Summary [file 41467_2022_30008_MOESM11_ESM.pdf]

nature research

Corresponding author(s): Ravid Straussman

Last updated by author(s): Sep 24, 2021

Reporting Summary

Nature Research wishes to improve the reproducibility of the work that we publish. This form provides structure for consistency and transparency in reporting. For further information on Nature Research policies, see our Editorial Policies and the Editorial Policy Checklist.

Statistics

For all statistical analyses, confirm that the following items are present in the figure legend, table legend, main text, or Methods section.

n/a	Confirmed	
☐	☒	The exact sample size (n) for each experimental group/condition, given as a discrete number and unit of measurement
☐	☒	A statement on whether measurements were taken from distinct samples or whether the same sample was measured repeatedly
☐	☒	The statistical test(s) used AND whether they are one- or two-sided<br>*Only common tests should be described solely by name; describe more complex techniques in the Methods section.*
☒	☐	A description of all covariates tested
☐	☒	A description of any assumptions or corrections, such as tests of normality and adjustment for multiple comparisons
☐	☒	A full description of the statistical parameters including central tendency (e.g. means) or other basic estimates (e.g. regression coefficient) AND variation (e.g. standard deviation) or associated estimates of uncertainty (e.g. confidence intervals)
☐	☒	For null hypothesis testing, the test statistic (e.g. F, t, r) with confidence intervals, effect sizes, degrees of freedom and P value noted<br>*Give P values as exact values whenever suitable.*
☒	☐	For Bayesian analysis, information on the choice of priors and Markov chain Monte Carlo settings
☐	☒	For hierarchical and complex designs, identification of the appropriate level for tests and full reporting of outcomes
☐	☒	Estimates of effect sizes (e.g. Cohen's d, Pearson's r), indicating how they were calculated

Our web collection on statistics for biologists contains articles on many of the points above.

Software and code

Policy information about availability of computer code

Data collection	Cells were imaged with the Operetta CLSTM High Content 10 Imaging system (Perkin Elmer) using 10x high NA objective
Data analysis	Image analysis and feature extraction were done using CellProfiler (Version 2.2.0). To determine the visual barcode identity of each cell, we used CellProfiler Analyst (Version 2.0) classifier supervised machine learning software.<br>Data analysis and statistical tests were performed using R (R version 3.6.0) and RStudio (Version 1.2.5033). Plotting the data was done using the ggplot2 package (version 3.3.0). Heatmaps were created using the pheatmap package ((version 1.0.10) Correlation plots were created using ggcorrplot (version 0.1.3) . Kolmogorov-Smirnov test as well as other statistical tests were performed using the stats package (version 3.6.0). Western blot analysis was done using Image Studio (version 5.2.5).

For manuscripts utilizing custom algorithms or software that are central to the research but not yet described in published literature, software must be made available to editors and reviewers. We strongly encourage code deposition in a community repository (e.g. GitHub). See the Nature Research guidelines for submitting code & software for further information.

Data

Policy information about availability of data

All manuscripts must include a data availability statement. This statement should provide the following information, where applicable:

- Accession codes, unique identifiers, or web links for publicly available datasets
- A list of figures that have associated raw data
- A description of any restrictions on data availability

All data generated or analyzed during this study are included in this published article and its supplementary information files. The different datasets that were used for RAR//RXR patient stratification are publicly available as follows: (a) Cirenajwis et al. dataset is available in GEO

under the accession number: GSE65904; (b) Budden et al. dataset is available in GEO under the
accession number: GSE59455; (c) Human melanoma tumors from TCGA were downloaded from
the GDC data portal https://portal.gdc.cancer.gov/. All the data relating to the TCGA protein analysis was downloaded from https://tcpaportal.org/. For the Protein-protein interactions network analyzed we used https://string-db.org/.

Field-specific reporting

Please select the one below that is the best fit for your research. If you are not sure, read the appropriate sections before making your selection.

☒ Life sciences ☐ Behavioural & social sciences ☐ Ecological, evolutionary & environmental sciences

For a reference copy of the document with all sections, see nature.com/documents/nr-reporting-summary-flat.pdf

Life sciences study design

All studies must disclose on these points even when the disclosure is negative.

Sample size	Sample size are depicted separately for every experiment in the text and sup figures.
Data exclusions	No data was excluded from the analysis
Replication	Both technical and biological replications were done. The number of the technical and biological repeats are depicted in the text and in the legend for each figure and sup. figure separately.
Randomization	n/a
Blinding	n/a

Reporting for specific materials, systems and methods

We require information from authors about some types of materials, experimental systems and methods used in many studies. Here, indicate whether each material, system or method listed is relevant to your study. If you are not sure if a list item applies to your research, read the appropriate section before selecting a response.

Materials & experimental systems

n/a	Involved in the study	
☐	☒	Antibodies
☐	☒	Eukaryotic cell lines
☒	☐	Palaeontology and archaeology
☐	☒	Animals and other organisms
☒	☐	Human research participants
☒	☐	Clinical data
☒	☐	Dual use research of concern

Methods

n/a	Involved in the study	
☒	☐	ChIP-seq
☐	☒	Flow cytometry
☒	☐	MRI-based neuroimaging

Antibodies

Antibodies used

1. pp65 Cell Signaling #3030 1:1000
2. YAP/TAZ Cell Signaling #8418 1:1000
3. p-YAP/TAZ Cell Signaling #4911 1:1000
4. AKT Cell Signaling #2920 1:1000
5. pAKT Cell Signaling #4060 1:1000
6. p53 Cell Signaling #48818 1:1000
7. JNK Cell Signaling #9251 1:1000
8. p-JNK Cell Signaling #3708 1:1000
9. pERK1/2 Cell Signaling #4370 1:1000
10. ERK1/2 Cell Signaling #9107 1:1000
11. p-CREB Cell Signaling #9198 1:1000
12. GAPDH Cell Signaling #2118 1:1000

Validation

All antibodies used are commercially available.
validation details can be found at:
1. https://www.cellsignal.com/products/primary-antibodies/phospho-nf-kb-p65-ser536-93h1-rabbit-mab/3033
2. https://www.cellsignal.com/products/primary-antibodies/yap-taz-d24e4-rabbit-mab/8418?site-search-type=Products&N=4294956287&Ntt=%238418&fromPage=plp&_requestid=1257429

3. https://www.cellsignal.com/products/primary-antibodies/phospho-yap-ser127-antibody/4911?site-search-type=Products&N=4294956287&Ntt=%234911&fromPage=plp&_requestid=1257456
4. https://www.cellsignal.com/products/primary-antibodies/akt-pan-40d4-mouse-mab/2920?site-search-type=Products&N=4294956287&Ntt=%232920&fromPage=plp&_requestid=1257486
5. https://www.cellsignal.com/products/primary-antibodies/phospho-akt-ser473-d9e-xp-rabbit-mab/4060?site-search-type=Products&N=4294956287&Ntt=%234060&fromPage=plp&_requestid=1257529
6. https://www.cellsignal.com/products/primary-antibodies/p53-do-7-mouse-mab/48818?site-search-type=Products&N=4294956287&Ntt=%2348818&fromPage=plp&_requestid=1257582
7. https://www.cellsignal.com/products/primary-antibodies/phospho-sapk-jnk-thr183-tyr185-antibody/9251?site-search-type=Products&N=4294956287&Ntt=%239251&fromPage=plp&_requestid=1257670
8. https://www.cellsignal.com/products/primary-antibodies/jnk1-2c6-mouse-mab/3708?site-search-type=Products&N=4294956287&Ntt=%233708&fromPage=plp&_requestid=1257736
9. https://www.cellsignal.com/products/primary-antibodies/phospho-p44-42-mapk-erk1-2-thr202-tyr204-d13-14-4e-xp-rabbit-mab/4370?site-search-type=Products&N=4294956287&Ntt=%234370&fromPage=plp&_requestid=1257791
10. https://www.cellsignal.com/products/primary-antibodies/p44-42-mapk-erk1-2-3a7-mouse-mab/9107?site-search-type=Products&N=4294956287&Ntt=%239107&fromPage=plp&_requestid=1257839
11. https://www.cellsignal.com/products/primary-antibodies/phospho-creb-ser133-87g3-rabbit-mab/9198?site-search-type=Products&N=4294956287&Ntt=%239198&fromPage=plp&_requestid=1257874
12. https://www.cellsignal.com/products/primary-antibodies/gapdh-14c10-rabbit-mab/2118?site-search-type=Products&N=4294956287&Ntt=%232118&fromPage=plp&_requestid=1257916

Eukaryotic cell lines

Policy information about cell lines

Cell line source(s)	A375 (ATCC ,CRL-1619), SK-Mel-5 (ATCC, HTB-70), HeLa (ATCC, CRM-CCL-2), RPE1 (ATCC, CRL-4000), HEK293T (ATCC, CRL-3216) were obtained from the ATCC. PC9 was a gift from Dr. Channing Yu of the Broad Institute of Harvard and MIT,
Authentication	Non of the cell line used were authenticated by us
Mycoplasma contamination	All cell lines were tested negative for Mycoplasma by PCR
Commonly misidentified lines (See ICLAC register)	No commonly misidentified cell lines was used in this study

Animals and other organisms

Policy information about studies involving animals; ARRIVE guidelines recommended for reporting animal research

Laboratory animals	5 weeks old female nude mice were used as detailed in the method section
Wild animals	No wild animals were used in this study
Field-collected samples	No field collected samples were used in the study
Ethics oversight	Mice studies were approved by the institutional animal care and use committee of the Weizmann institute (00400120-3)

Note that full information on the approval of the study protocol must also be provided in the manuscript.

Flow Cytometry

Plots

Confirm that:

- [x] The axis labels state the marker and fluorochrome used (e.g. CD4-FITC).
- [x] The axis scales are clearly visible. Include numbers along axes only for bottom left plot of group (a 'group' is an analysis of identical markers).
- [x] All plots are contour plots with outliers or pseudocolor plots.
- [x] A numerical value for number of cells or percentage (with statistics) is provided.

Methodology

Sample preparation	ImageStream was used to image the visual barcodes after tumor extraction as detailed in the method section.
Instrument	ImageStreamX Mark II, AMNIS corp
Software	IDEAS 6.2
Cell population abundance	*Describe the abundance of the relevant cell populations within post-sort fractions, providing details on the purity of the samples and how it was determined.*

Gating strategy

Describe the gating strategy used for all relevant experiments, specifying the preliminary FSC/SSC gates of the starting cell population, indicating where boundaries between "positive" and "negative" staining cell populations are defined.

☒ Tick this box to confirm that a figure exemplifying the gating strategy is provided in the Supplementary Information.
